# Using Commercial Bio-Functional Fungal Polysaccharides to Construct Emulsion Systems by Associating with SPI

**DOI:** 10.3390/foods14020215

**Published:** 2025-01-12

**Authors:** Laixin Dai, Qingfu Wang, Lining Wang, Qinghua Huang, Biao Hu

**Affiliations:** Guangdong Engineering Laboratory of Biomass High-Value Utilization, Institute of Biological and Medical Engineering, Guangdong Academy of Sciences, Guangzhou 510316, China

**Keywords:** commercial fungi polysaccharides, soy protein isolate, complex formation, emulsion

## Abstract

Fungi polysaccharides are nutraceutical-rich compounds with bioactive properties, offering promising applications in food formulation. This study examined the non-covalent complexation of commercial polysaccharides derived from the fruiting bodies of *Auricularia auricula-judae* (AA) and *Ganoderma lucidum* (GL) and soy protein isolate to enhance emulsifying properties. Complexes were examined across protein-to-polysaccharide ratios (0:1 to 1:0), pH levels (3 to 7), and heat treatment conditions. Results indicated a maximum insoluble association at pH 4 for both SPI-AAP and SPI-GLP complexes, with SPI-AAP complexes remaining soluble at pH 3, while SPI-GLP complexes exhibited insolubility. Heat treatment had a limited effect on electrostatically driven complexation but resulted in larger particles through a protein-denaturation-induced increase of hydrophobic interactions. In terms of emulsifying properties, individual GLPs demonstrated superior performance compared to individual AAPs. The GLPs engaged in competitive adsorption at the oil–water interface alongside SPI, resulting in larger emulsion droplet sizes compared to either component alone. The association of either AAPs or GLPs with SPI enhanced the emulsion stability against coalescence and Ostwald ripening. Commercial fungal polysaccharides demonstrate substantial potential for incorporation into manufactured food products, particularly in colloidal formulations.

## 1. Introduction

Polysaccharides, composed of multiple monosaccharides, represent a crucial class of bioactive biopolymers found in various organisms, including plants, bacteria, seaweeds, and fungi [1]. Natural polysaccharides are recognized for their beneficial effects and are widely utilized in pharmaceuticals, food, healthcare, and cosmetics [2,3]. Their biological activities encompass anti-tumor, anti-inflammatory, immunomodulatory, antioxidant, and anti-aging effects [4,5,6]. Additionally, the high viscosity, gelling properties, and water-holding capacity of polysaccharides make them valuable as thickeners, stabilizers, and texturizers in various applications [7,8,9].

Among natural polysaccharides, those derived from edible and medicinal fungi, such as *Auricularia auricula-judae* (AA) and *Ganoderma lucidum* (GL), have gained attention due to the increasing interest in healthy diets and sustainable resources. The AA mushroom, commonly known as Jew’s ear, black wood ear, or jelly ear, is the third most widely cultivated edible fungus globally and is prevalent in many oriental Asian countries [10,11]. GL, known as “*Lingzhi*” in Chinese, has been highly regarded as a valuable herbal medicine across Asia for thousands of years [12]. Polysaccharides are the key bioactive components in the fruiting bodies of AA and GL, exhibiting a molecular weight distribution ranging from 10^3^ to 10^6^ Da. AA polysaccharides (AAPs) are predominantly heteropolysaccharides with complex and diverse structures [13,14]. Previous research has shown that the fundamental structure of AAPs is primarily linked by (1→3), (1→4), and (1→6) glycosidic linkages, with branches formed by (1→6), (1→4, 6), and (1→2) linkages, comprising neutral sugars (e.g., glucose, mannose, galactose, xylose, arabinose, rhamnose) and sugar derivatives (e.g., glucuronic acid, galacturonic acid, glucosamine) [15,16]. Similarly, GL polysaccharides (GLPs) display a high degree of molecular diversity, with over 200 hetero-polysaccharides characterized by various glycosidic linkages [17].

The differences in the molecular characteristics of AAPs and GLPs arise from variations in the source fungi, extraction, purification, and analytical methods. Consequently, these molecular variations influence their physicochemical properties, biological activities, and techno-functional properties. Current research on AAPs and GLPs predominantly focuses on their extraction, purification, and bioactivities, with a strong emphasis on high-refinement processes for pharmaceutical applications or functional foods. However, the application of these fungal polysaccharides in food systems remains limited, particularly as raw food materials which contribute to food texture and the construction of food systems on an industrial scale. Commercial fungal polysaccharides extracted using cost-effective, large-scale methods may contain impurities and heterogeneous compositions, resulting in functional properties that differ from those of laboratory-extracted counterparts, such as reduced viscosity.

This study investigates the potential of utilizing polysaccharides from AA and GL to construct emulsion systems in association with soy protein isolate (SPI). The combination of protein–polysaccharide complexes is of significant interest for producing emulsion droplets suitable for encapsulation, delivery, and the creation of novel food structures [18,19]. Proteins possess excellent emulsifying properties that facilitate the formation of small emulsion droplets due to their amphiphilic nature, wherein the hydrophobic regions interact with the oil phase while the hydrophilic regions remain in the aqueous phase, stabilizing the emulsion through electrostatic and steric repulsion [20,21]. However, emulsions stabilized by proteins are susceptible to variations in pH, ionic strength, concentration, and heat treatments, which can induce protein denaturation [21,22,23]. The association with polysaccharides allows for the design of non-adsorbing polysaccharide regions around emulsion droplets, enhancing steric stabilization [24]. This approach presents potential benefits for improving food healthiness by incorporating protein–polysaccharide complexes into nutrient delivery systems, encapsulation active ingredients, and the protection of bioactive components when considering the design of colloidal food structures.

**Scheme 1:**
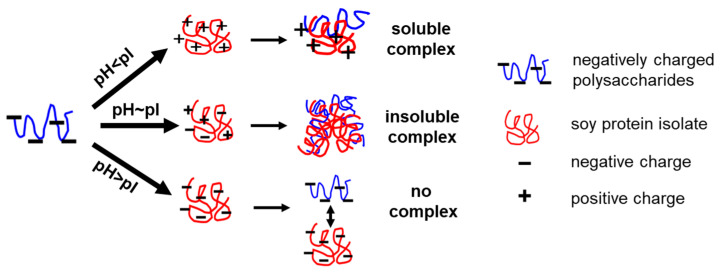
Schematic diagram of electrostatic interaction between polysaccharides and proteins.

Noncovalent interactions between food proteins and polysaccharides are primarily driven by electrostatic forces, with hydrogen bonding and hydrophobic interactions also contributing to the formation of protein–polysaccharide complexes [25]. Consequently, the ratio of biopolymers, pH, and temperature influences the coacervation behavior of these complexes. Under varying conditions, protein–polysaccharide complexes may exist in either a single-phase system (co-soluble without interaction or as a soluble complex) or a two-phase system (phase separation of non-interacting biopolymers or as an insoluble complex) as shown in Scheme 1 [19,26]. For instance, SPI exhibits both positive and negative charges at pH levels below and above 4.5, respectively. In contrast, fungal polysaccharides (AAPs and GLPs) are negatively charged within the pH range of 3 to 7, encompassing the typical pH values encountered in most food systems. It is hypothesized that SPI-AAP and SPI-GLP complexes will begin to generate and reach the isoelectric point of the biopolymer system at certain pH values which vary with protein and fungi polysaccharides ratio. With the establishment of SPI-AAP and SPI-GLP complexes, an improvement in emulsifying properties is anticipated, resulting in smaller emulsion droplets compared to individual polysaccharides, as well as enhanced emulsion stability against droplet coalescence in comparison to individual proteins.

## 2. Materials and Methods

### 2.1. Materials

Soy protein isolate was purchased from Wanbang Chemical Technology Co., Ltd. (Zhengzhou, China). Commercial polysaccharides derived from *Auricularia Auricula-judge* and *Ganoderma lucidum* were obtained from Shaanxi Ivy Bioengineering Co., LTD. (Xi’an, China), with compositions of 79.4 ± 1.7% and 75.7 ± 1.6% crude polysaccharides, 4.8 ± 0.1% and 5.6 ± 0.3% moisture, 2.0 ± 0.0% and 3.7 ± 0.1% protein, and 1.1 ± 0.0% and 2.7 ± 0.2% ash, respectively. Soy oil was procured from Yihai Kerry (Shanghai, China). Hydrochloric acid and sodium hydroxide (purity ≥ 96%) were supplied by the Guangzhou Chemical Reagent Factory (Guangzhou, China). Sodium dodecyl sulfate ((SDS, purity ≥ 99%) was purchased from Macklin Inc. (Shanghai, China).

### 2.2. Preparation of Soy Protein Isolate–Polysaccharide Complexes

SPI, AAPs, and GLPs at a concentration of 3% (*w*/*w*) were dissolved in water containing 0.05% of the antibacterial agent ProClean 950 (Beyotime Biotechnology, Shanghai, China). The SPI solution was mixed for 3 h, while the polysaccharide solutions were stirred for 1 h at room temperature. The formation of the SPI–polysaccharide complexes was investigated under varying biopolymer mixing ratios, pH conditions, and heat treatments. Mixtures with a total biopolymer concentration of 3% (*w*/*w*) were prepared at SPI-to-polysaccharide ratios of 0:1, 0.5:1, 1:1, 2:1, 4:1, 8:1, and 1:0, and stirred for 10 min. The pH of the mixtures was adjusted to 3.0, 4.0, 5.0, 6.0, and 7.0. Heat treatment was applied by incubating the samples at 90 °C for 20 min, followed by cooling to room temperature for further analysis.

### 2.3. Emulsion Preparation

Oil-in-water emulsions were prepared using SPI–polysaccharide complexes at selected SPI-to-polysaccharide ratios of 0:1, 1:1, 2:1, 4:1, and 1:0 at pH 3 and 4. The complexes acted as emulsifying agents and were mixed with 10% (*w*/*w*) oil using an Ultra Turrax homogenizer (IKA T25 digital, IKA-Werke GmbH & CO.KG, Staufen, Germany) at 10,000 rpm for 3 min. An amount of 50 mL of coarse emulsion was then further homogenized at 50 MPa for six passes using an EmulsiFlex-C5 homogenizer (Avestin, Inc., Ottawa, ON, Canada) in an ice-water bath to maintain the sample temperature.

### 2.4. Optical Observation

A 5 mL aliquot of the SPI–polysaccharide complexes or emulsions was transferred into glass tubes and stored still at 4 °C overnight. Images of the samples were captured using a smartphone camera (Honor 70pro, Honor Device Co., Ltd., Shenzhen, China). The microstructure of the mixtures at various ratios and pH levels was observed using an optical microscope (Olympus CKX53, Olympus, Tokyo, Japan) equipped with a PLN2X objective lens at 40 × magnification.

### 2.5. Turbidity Measurement

The turbidity of the complexes was determined by measuring their absorbance at 600 nm using a spectrophotometer (Shimadzu UV-2600i, Kyoto, Japan). The samples were diluted to a polymer concentration of 0.5% with distilled water adjusted to the relevant pH. Distilled water served as the blank.

### 2.6. Particle Size Analysis

The particle size distribution and mean volume diameters (*d*_43_) of the mixtures and emulsions were measured using a light scattering particle size analyzer (OMEC LS-POP (9)/609, OMEC Instruments Co., Ltd., Zhuhai, China). A volume of 400 mL of pH-adjusted water was added to the sample chamber with an arm stirrer set to 2000 rpm. An appropriate amount of the sample was introduced to achieve an obscuration ratio within the 10–20% range, ensuring minimal multiple scattering. The refractive index for the biopolymer mixtures and emulsions was set to 1.52, while 1.33 was used for the dispersant.

### 2.7. ζ-Potential Measurement

The ζ-potential of the SPI–polysaccharide complexes and emulsions was measured to assess surface charge characteristics. Samples were diluted to a polymer concentration of 0.15%, and 0.9 mL was loaded into cuvettes (Omega Cuvettes, Anton Paar GmbH, Graz, Austria). Using an electrophoretic light-scattering method with a zeta-sizer (LITESIZER 500, Anton Paar GmbH, Graz, Austria), the electrophoretic mobility of the samples was measured, and their ζ-potential was calculated following the Smoluchowski approximation.

### 2.8. Statistical Analysis

All measurements were conducted in triplicate using freshly prepared samples in duplicate. Data were processed to obtain means and standard deviations using Re (Microsoft Corporation, Redmond, WA, USA) and visualized in figures created with Origin software (OriginLab, Northampton, MA, USA).

## 3. Results and Discussion

### 3.1. Formation of Soy Protein Isolate–Polysaccharide Complexes

**Figure 1:**
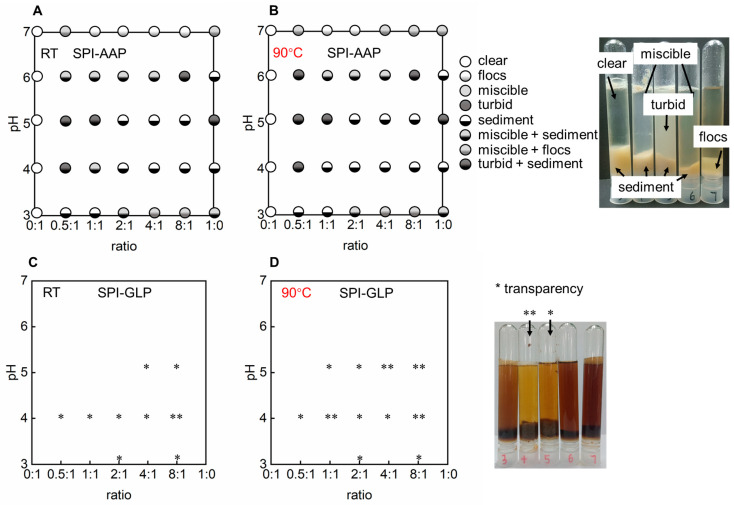
The phase diagram of complexes of soy protein isolate (SPI) and polysaccharides derived from *Auricularia auricula-judae* (AAP) (**A**,**B**) and *Ganoderma lucidum* (GLP) (**C**,**D**) at 25 °C and 90 °C.* means the sample present a higher transparency and ** means the highest transparency amongst a set of samples.

This section examines the conditions required for polysaccharides to form complexes with SPI, analyzing different SPI-to-polysaccharide ratios and pH levels both at room temperature and after heating at 90 °C. Schematic phase diagrams were generated to summarize the complexation behavior of SPI with AAP and GLP, using visual observations and optical microscopy. Phase diagrams for the SPI-AAP system were constructed based on the transparency and compactness of complex clusters as shown by visual inspection and microscopy (Figure S1). Five primary states were identified: (1) clear, indicating high transparency; (2) flocs, signifying gravitational phase separation, exhibiting higher transparency than sediment and appearing clear or with subtle particles under the microscope; (3) miscible, denoting stable semi-transparent dispersions; (4) turbid, indicating extremely low transparency with observable particles under a microscope; (5) sediment, formed by large polymer aggregates settling at the bottom of the tube under gravity, exhibiting very low transparency and being visible under a microscope (Figure 1A,B). Combined states, such as miscible + sediment, miscible + flocs, and turbid + sediment were also observed (Figure 1A,B). For the SPI-GLP complex, phase diagrams were based on visual transparency due to the dark color of GLP, complicating appearance differentiation between miscible, turbid, flocs, and sediment states (Figure S1 and Figure 1C,D). The transparency was compared across different pH levels at constant SPI-to-polysaccharide ratios.

**Figure 2:**
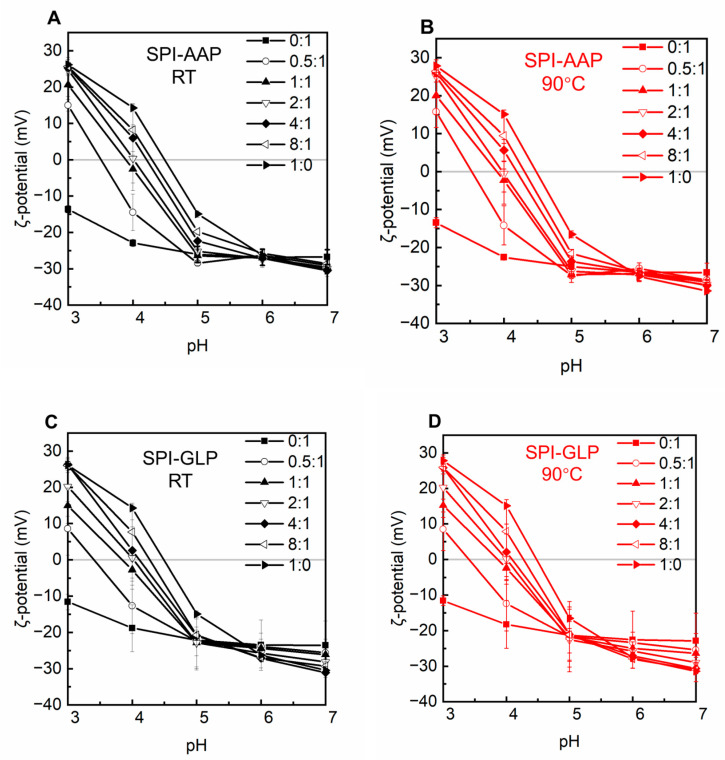
The ζ-potential of SPI-AAP (**A**,**B**) and SPI-GLP (**C**,**D**) complexes at 25 °C (in black) and 90 °C (in red).

**Figure 3:**
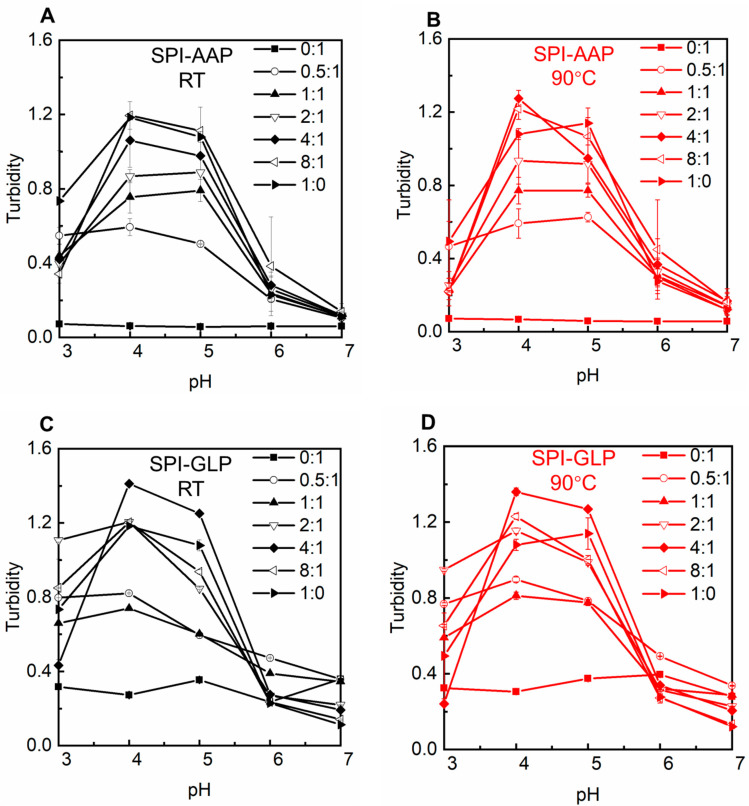
The turbidity of SPI-AAP (**A**,**B**) and SPI-GLP (**C**,**D**) complexes at 25 °C (in black) and 90 °C (in red).

Visual observation and microscopic analysis revealed that AAP solutions remained clear across all tested pH levels, indicating stability against pH variation. Similarly, GLP exhibited no phase separation or transparency changes, suggesting stability under varying pH conditions (Figure 1). This stability was consistent with ζ-potential measurements for AAP and GLP, which remained constant from pH 4 to 7, albeit with a shift in ζ-potential from approximately −20 mV to −10 mV (Figure 2). Individual SPI formed sediment at pH 4 to 6, near its isoelectric point (pH 4.5) (Figure 1 and Figure 2). At the isoelectric point, proteins aggregate due to strong electrostatic attraction, resulting in large, insoluble particles and phase separation. Away from the isoelectric point, SPI carries a consistent electric charge, promoting electrostatic repulsion to keep the proteins apart and resulting in a loose sediment structure at pH 3 and a miscible state at pH 7 (Figure 1). Turbidity measurements of individual polysaccharides and SPI supported these findings (Figure 3). The turbidity of AAP remains constant at around 0.1, while GLP fluctuated between 0.2, and 0.4. SPI had the highest turbidity at pH 4 and 5 with values of 1.2 ± 0.0 and 1.1 ± 0.0 which decreased to 0.1 ± 0.0 at pH 7 (Figure 3). The results indicated a higher aggregation near the isoelectric point and an increased solubility away from pH 4.5.

Electrostatic, hydrogen bonding, and hydrophobic interactions are the primary forces in protein–polysaccharide complexes. The behavior of polysaccharide and SPI mixtures is influenced by the intrinsic properties of the individual polymers, protein–polysaccharide ratios, and environmental factors such as pH and heat treatment [27]. The SPI-AAP mixtures exhibited sediment formation at pH 4 and 5 while forming miscible, flocs, or floc + miscible phases at pH 7 across all mixing ratios (Figure 1A). According to Figure 1C, all of the SPI-GLP mixtures formed aggregates at pH 4 and displayed increased transparency of the aqueous phase due to electrostatic interactions. At higher pH levels (6 and 7), the SPI-GLP complex had the darkest color across tested ratios, while both SPI-AAP and SPI-GLP complexes exhibited the lowest turbidity at pH 7 (Figure 3). The influence of pH on electrostatic interactions in protein–polysaccharide complexes is evident, as protein surface charges vary with pH due to the ionizable amino acids on the solvent-exposed surfaces of proteins [28]. At pH 7, the negative charges on both proteins and polysaccharides created repulsion, preventing complex association [29]. At lower pH levels (pH 6 and 5), despite an overall negative protein charge, protonation of partial amino groups led to positively charged patches that could interact with negatively charged polysaccharides. Consequently, mixtures at pH 4 and 5 exhibited the highest turbidity, indicative of protein–protein aggregates and protein–polysaccharide complexes (Figure 2 and Figure 3).

At protein-to-polysaccharide ratios of 0.5:1, 1:1, and 2:1, SPI-AAP mixture at pH 3 formed sedimented complexes, while higher protein content (ratios of 4:1 and 8:1) produced miscible or flocculated phases (Figure 1A). This behavior aligned with ζ-potential measurements, showing that the pH at which surface charge equaled zero shifted from 4.3 to 3.5 as the protein-to-polysaccharide ratio decreased from 8:1 to 0.5:1 (Figure 2A). Therefore, pH 3 was closer to the isoelectric point of SPI-AAP mixtures at ratios of 0.5:1–2:1 compared to higher ratios of 4:1 and 8:1. This can be attributed to the higher content of polysaccharides in the mixture, where more positively charged patches on the protein assembled with negatively charged regions on the polysaccharides, reducing the overall surface charge of the SPI-AAP. For example, the ζ-potential of SPI-AAP mixtures decreased from 14.3 ± 0.8 mV (pure protein, 1:0 ratio) to near zero at the ratio of 2:1 and eventually reached a negative value of −14.5 ± 5.0 mV (at the ratio of 0:1) (Figure 2A). These results demonstrate that AAP binding occupied the positively charged areas on the proteins.

A similar pattern was observed for SPI-GLP, where the ζ-potential of the mixtures decreased at pH 5 and 3, becoming zero, then turning to negative at pH 4 as GLP content increased (Figure 2C). Additionally, protein self-aggregation at its isoelectric point contributes to the formation of more particles [30]. This trend was evident in SPI-AAP or SPI-GLP mixtures with higher protein contents (ratios of 4:1 and 8:1), where complete phase separation occurred at pH 5 (close to the isoelectric point of SPI, at pH 4.5), resulting in a transparent upper aqueous phase (Figure 1).

**Figure 4:**
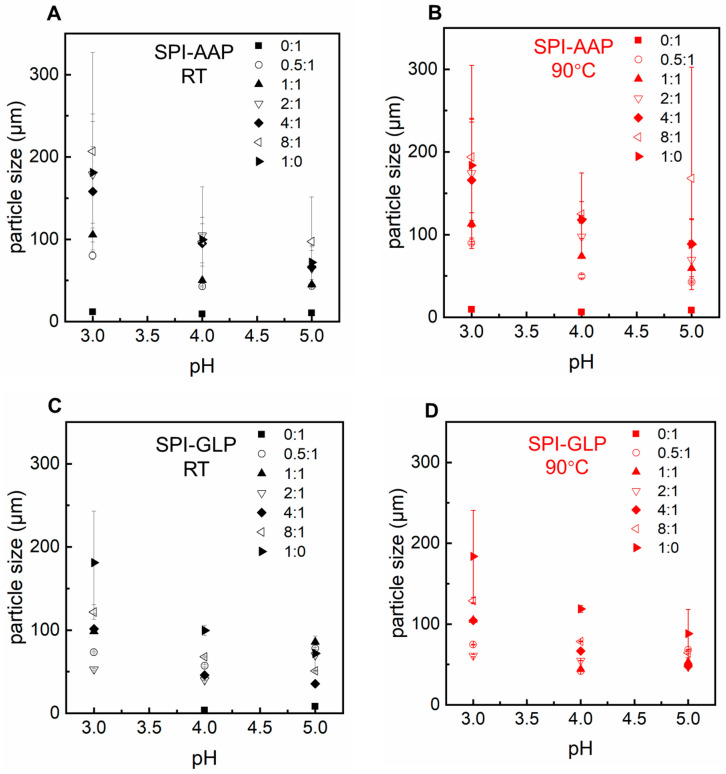
The particle size of SPI-AAP (**A**,**B**) and SPI-GLP (**C**,**D**) complexes at 25 °C (in black) and 90 °C (in red) at pH 3 to 5.

Particle size measurement was conducted for the mixtures at pH levels where the protein and polysaccharides were oppositely charged (pH 3–5, Figure 4) since it has been reported that protein–polysaccharide complex formation is optimal at the pH of electrical equivalence of individual polymers [29,31]. The results indicate that larger particles were observed as the protein-to-polysaccharide ratio increased from 0.5:1 to 8:1 for both SPI-AAP and SPI-GLP mixtures (Figure 4). Lan et al. reported similar findings, demonstrating that mixtures with higher protein content tended to form insoluble biopolymer complexes [32]. However, the particle sizes of SPI-AAP and SPI-GLP complexes at pH 4 were larger than those observed at pH 4 and 5, contrasting with the microscopic results where fewer particles were detected at pH 3 (Figure 4 and Figure S1). This discrepancy could be due to the limitations of static light scattering measurements, which are based on Mie theory and are more accurate for spherical particles, potentially mispresenting the diameter of protein–polysaccharide complexes at pH 3 [29].

The impact of heat treatment, a common food processing method, was also evaluated for its effect on SPI-AAP and SPI-GLP complex formation. Thermal treatment at 90 °C showed negligible effects on the phase behavior and ζ-potential of SPI-AAP (Figure 1B and Figure 2B), indicating that electrostatic interactions are the dominant forces governing SPI-AAP complexation. However, for SPI-GLP complexes (Figure 1D), the aqueous phase at pH 5 became more transparent after heating, suggesting that thermal treatment intensified the sedimentation process within SPI-GLP complexes or the aggregation of protein. The turbidity of SPI-GLP mixtures at pH 5 post-heating was higher than that of untreated mixtures across all protein-to-polysaccharide ratios from 0.5:1 to 8:1 (Figure 3D). This increase in turbidity likely resulted from altered protein–protein interactions, leading to denaturation [33]. Alteration in hydrogen bonds, hydrophobic interactions, or van der Waals forces affected the 7S and 11S fractions of SPI, which denature at or below 90 °C [34].

### 3.2. Emulsifying Properties of Protein–Polysaccharide Complexes

Protein–polysaccharide complexes have garnered interest due to their high stability in preventing emulsion particles from merging caused by coalescence, Ostwald ripening, or aggregation, compared to individual protein or polysaccharides [35,36]. This enhanced stability is primarily attributed to the presence of polysaccharides that, when associated with proteins, increase steric and electrostatic repulsion through the formation of a thick interfacial layer in the aqueous phase, surrounding emulsifier-coated oil droplets [37]. The emulsifying properties of SPI-AAP and SPI-GLP complexes at selected ratios of 1:1, 2:1, and 4:1 were examined. Emulsions were prepared at pH 3 and pH 4, conditions under which SPI was negatively charged while AAP or GLP were considered optimal for protein–polysaccharide association.

**Figure 5:**
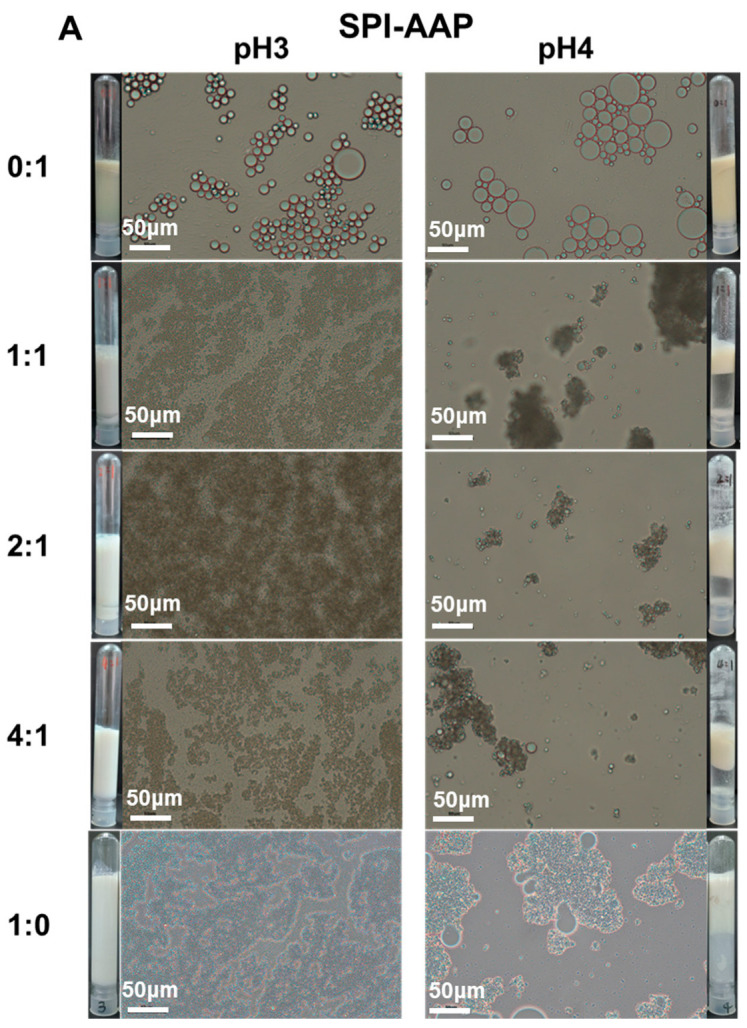

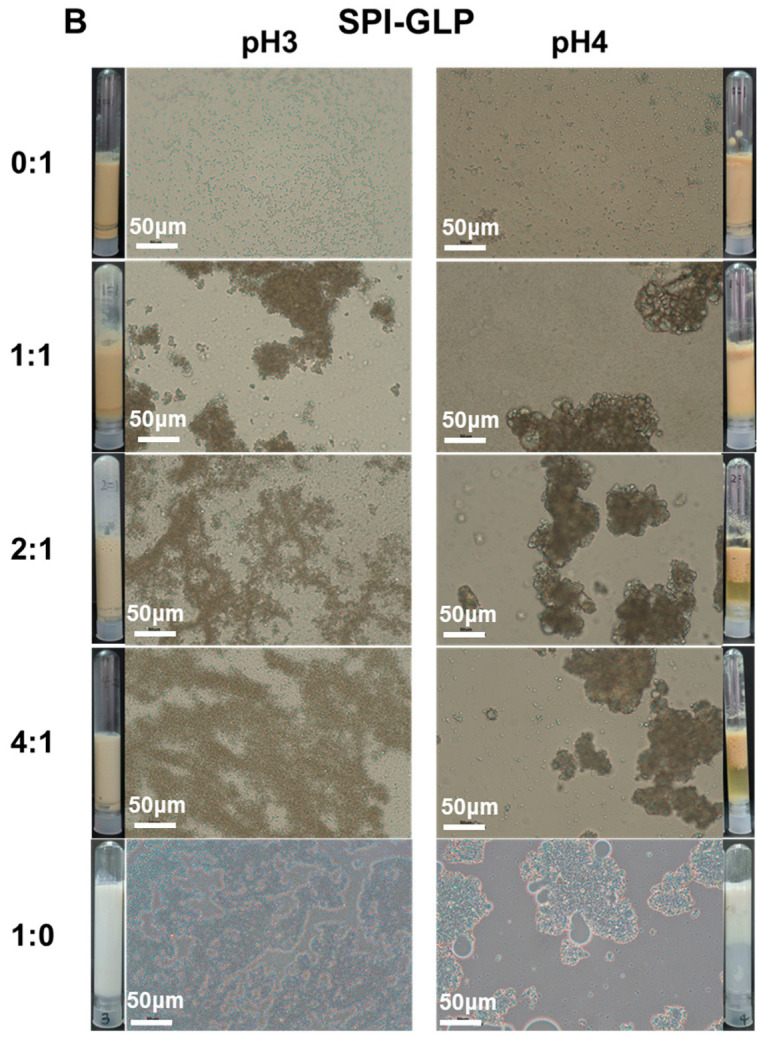
The appearance and optical microscopy images of emulsions prepared by SPI-AAP (**A**) and SPI-GLP (**B**) at ratios of 1:1, 2:1, and 4:1 at pH 3 and 4. The scale bar is 50 μm.

**Figure 6:**
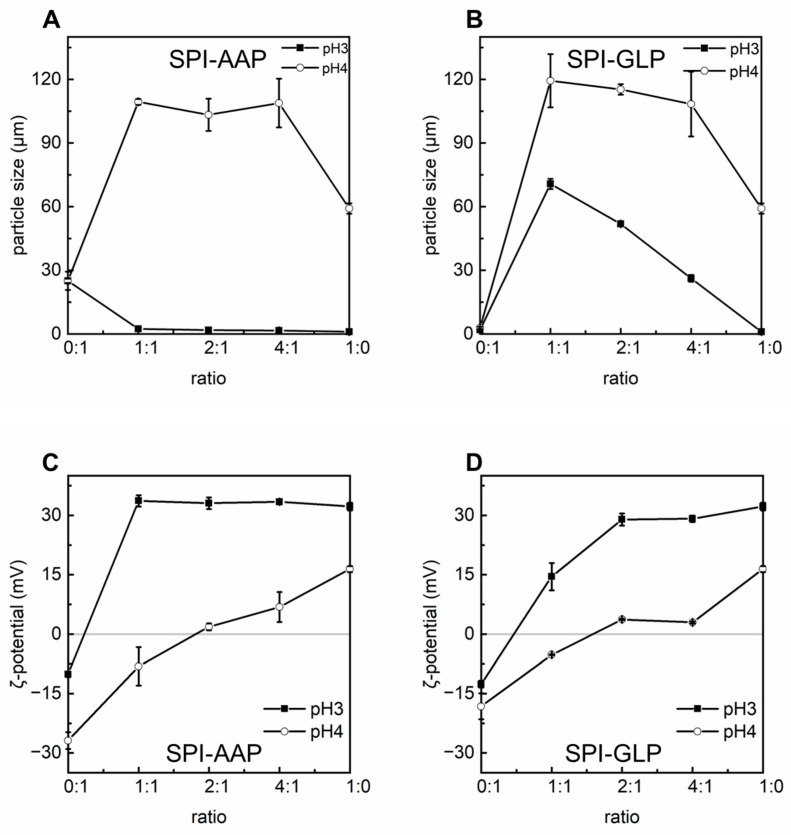
The particle size (*d*43, (**A**,**B**)) and ζ-potential (**C**,**D**) of emulsions prepared by SPI-AAP and SPI-GLP complexes at ratios of 1:1, 2:1, and 4:1 at pH 3 and 4.

Individually, AAP and GLP demonstrated the ability to produce emulsions with good dispersity at both pH 3 and pH 4 without emulsion droplet aggregation or phase separation (Figure 5). However, after overnight storage, emulsions prepared with AAP exhibited creaming, indicating limited stability as emulsion droplets merged together into larger ones that eventually ruptured, releasing internal oil. Emulsions stabilized by SPI-AAP and SPI-GLP complexes and individual SPI displayed a homogenous appearance at pH 3; whereas at pH 4, phase separation was observed, suggesting lower dispersity (Figure 5), which is attributed to electrostatic attraction between the emulsion particles surrounded by protein–polysaccharide complexes or proteins. This observation highlights the substantial influence of pH on the stability of emulsions, where the sensitivity of proteins to environmental pH changes plays a critical role. The particle size measurements of the emulsions aligned with these findings, as emulsions stabilized by SPI-AAP complexes exhibited larger particle sizes at pH 4 (~105 μm) compared to pH 3 (~2 μm) (Figure 6A).

Microscopic analysis further revealed greater coalescence of emulsion droplets at pH 4, resulting from aggregation (Figure 5). Coalescence occurs when emulsion droplets approach each other during flocculation, leading to the thinning and eventual disruption of the liquid film between droplets. Notably, emulsions prepared with individual SPI exhibited more severe droplet merging than those stabilized by SPI-AAP or SPI-GLP complexes at all tested ratios at pH 4. The results indicated that the emulsion prepared by SPI-AAP or SPI-GLP complexes was more stable against coalescence or Ostwald ripening than individual SPI. The polysaccharides hinder the emulsion droplets from getting close to each other due to spatial site effects [38,39].

It is worth noting that emulsions stabilized by individual AAPs displayed larger particles compared to those stabilized by individual GLPs, implying the higher interfacial activity of GLP. The particle sizes of emulsions prepared with AAP and GLP at pH 3 are 25.0 ± 4.3 μm and 2.0 ± 0.3 μm, respectively (Figure 6A,B). Emulsification by shear homogenization is a thermodynamically unstable process where the Ultra Turrax device shears oil into small droplets dispersed in the aqueous phase. Emulsifiers then stabilize these droplets by diffusing to the oil–water interface, adsorbing with their hydrophobic regions into the oil phase, and structurally rearranging to achieve high surface coverage to prevent coalescence [18,40,41]. Emulsifiers with higher interfacial activity form smaller, more stable emulsion droplets. Certain polysaccharides can inherently act as emulsifiers due to either the hydrophobic side groups or surface-active proteins naturally attached to their backbones with physical or chemical interactions [39,42]. The measured protein content in GLP (3.7 ± 0.1%) could be one possible reason explaining its high interfacial activity. However, the specific interactions between GLP and its naturally contained protein remain unclear, which would be an interesting topic to be further explored in the future to establish GLP as a natural emulsifier for food applications.

At pH 3, the emulsions prepared using SPI-AAP complexes (2.4 ± 0.0 μm at a ratio of 1:1) had smaller particle sizes compared to those prepared with individual AAP (25.0 ± 4.3 μm), indicating that coacervation with SPI enhanced the emulsifying properties of AAP. For SPI-GLP complexes at pH 3, the particle size of emulsions decreased from 70.7 ± 2.4 μm to 51. 9 ± 0.5 μm and 26.2 ± 1.6 μm as the SPI-to-GLP ratio increased from 1:1 to 2:1, and 4:1, respectively (Figure 6). This suggests that an increased SPI content enhances the emulsifying properties of SPI-GLP complexes, resulting in smaller emulsion particles. However, these particle sizes were still larger than those of emulsions stabilized by individual GLPs (2.0 ± 0.3 μm), potentially due to the ζ-potential of SPI-GLP complexes being closer to zero, which promotes aggregation (Figure 6D).

**Scheme 2:**
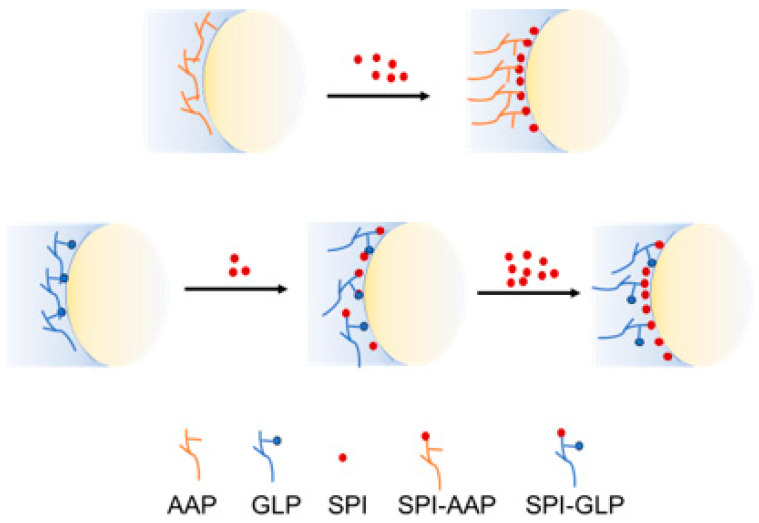
Schematic diagram of biopolymers distribution at oil–water interface.

Given the inherent interfacial activity of individual GLPs, it is proposed that GLPs compete with SPI for absorption at the oil–water interface, as illustrated in Scheme 2. At a lower SPI ratio (1:1) in the SPI-GLP complex, both SPI and GLPs can absorb at the oil–water interface partially exposing proteins to the aqueous phase. This arrangement suggests that emulsions prepared with SPI-GLP are more susceptible to pH-induced electrostatic interaction changes due to the exposed proteins being more sensitive. Conversely, at higher SPI contents, proteins dominate the interface while AAP occupies the outer layer conferring stability. Increased protein content enhances electrostatic repulsion due to higher ζ-potential and shifts the balance, with polysaccharides positioning themselves on the exterior to further stabilize the emulsions through steric effects.

## 4. Conclusions

This study investigated the association behavior of SPI with commercially available bio-functional fungal polysaccharides, showing that electrostatic interactions predominantly drive the SPI-AAP and SPI-GLP complexes, particularly at pH 3 and 4, where both SPI and polysaccharides are negatively charged. At pH 3, SPI-AAP complexes displayed higher solubility and lower turbidity, while SPI-GLP complexes performed similar behavior at pH 4. Optimal complexation occurred at pH 4 across SPI-to-polysaccharide ratios of 1:1, 2:1, and 4:1, where the ζ-potential approached neutrality and turbidity peaked. At these ratios, SPI-AAP complexes stabilized soybean oil, forming smaller emulsion droplets with enhanced stability against coalescence and Ostwald ripening compared to AAP alone. In contrast, SPI-GLP complexes demonstrated strong emulsifying capabilities but exhibited droplet aggregation due to competitive adsorption between SPI and GLP at the oil–water interface, reducing dispersity. This research underscores the potential of bio-functional polysaccharide–SPI complexes as emulsifiers to develop innovative food structures and encapsulation systems for bioactive compounds. Further investigation should focus on understanding the stability and emulsifying properties of these biopolymer complexes under various processing conditions and further examine the interfacial behavior of GLP to assess its viability as a natural emulsifier. Investigating the bioactivity of SPI-AAP and SPI-GLP complexes, their structure-function relationships, and functionality in various food systems could expand the applicability and market potential of commercially available bio-active polysaccharides. Furthermore, the association of polysaccharides may influence the encapsulation of bioactivities by soy protein isolate through self-assembly, an aspect that deserves further investigation [43].

## Data Availability

The original contributions presented in the study are included in the article, further inquiries can be directed to the corresponding authors.

## Supplementary Materials

The following supporting information can be downloaded at: https://www.mdpi.com/article/10.3390/foods14020215/s1, Figure S1: The appearance and optical microscopy images of soy protein isolate (SPI), polysaccharides derived from *Auricularia auricula-judae* (AAP) and *Ganoderma lucidum* (GLP), and their mixtures at ratios (r) of 0:1 to 1:0 at pH 3 to 7 at 25 °C and 90 °C. Figure S2: The particle size distribution of emulsions prepared by SPI-AAP and SPI-GLP complexes at ratios of 1:1, 2:1, and 4:1 at pH 3 (A) and 4 (B).
