# Supplementary figures and images for: Using Commercial Bio-Functional Fungal Polysaccharides to Construct Emulsion Systems by Associating with SPI
Source: Foods. 2025 Jan 12;14(2):215. doi: 10.3390/foods14020215 (PMC11764902; doi:10.3390/foods14020215)

Figure S1.

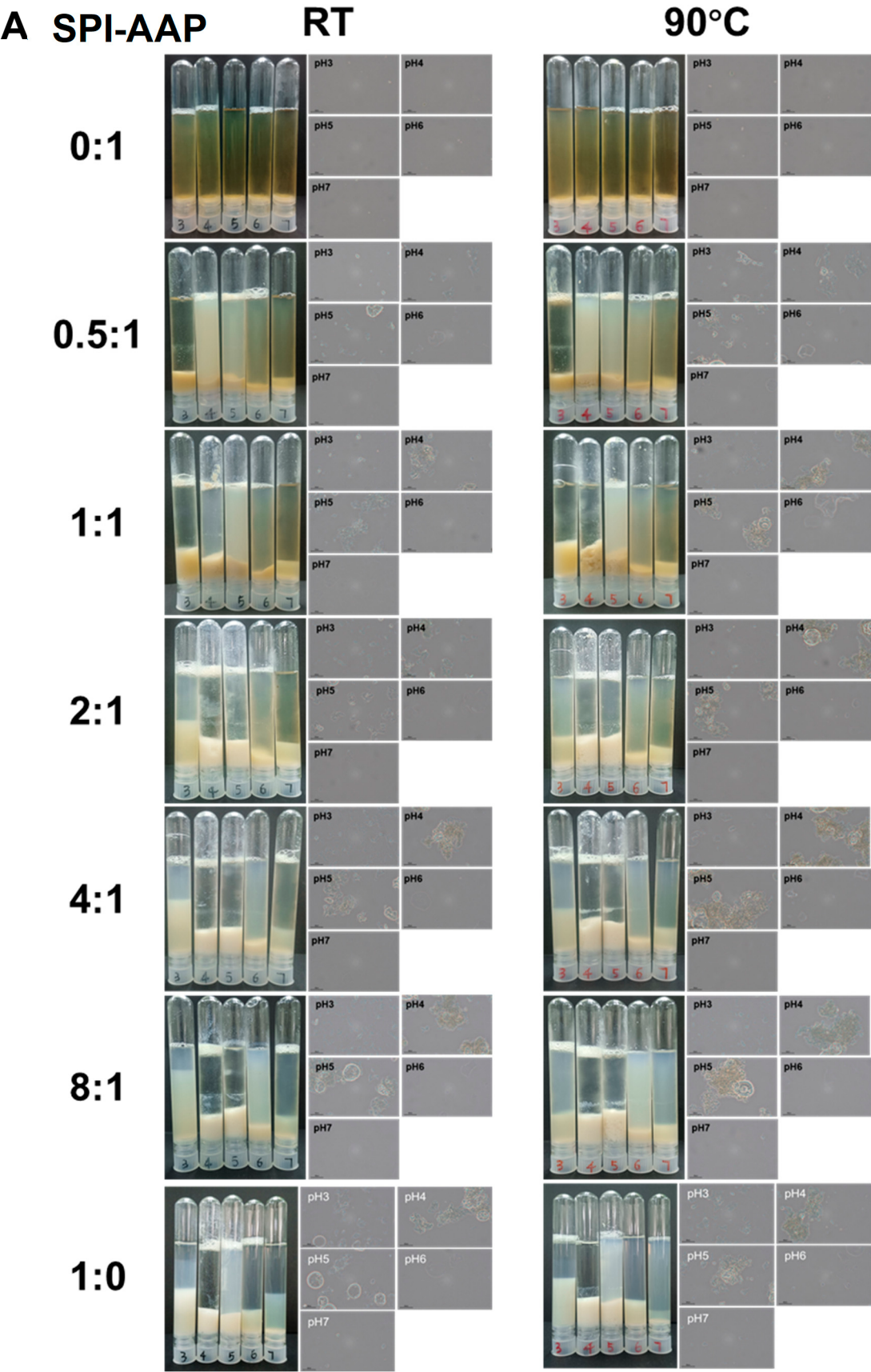



Figure S2.

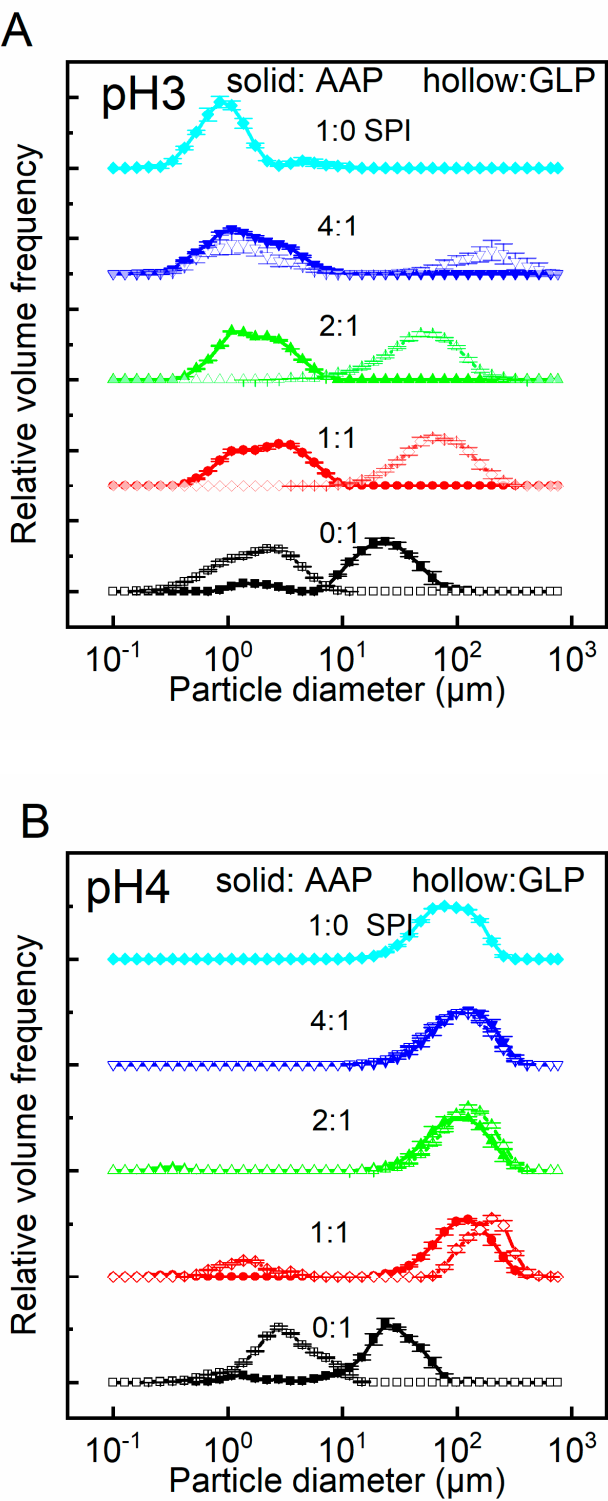

Supplement: Supplementary file 1 [file foods-14-00215-s001.zip › foods-3338648-supplementary.pdf]
